# Active control on switchable waveguide of elastic wave metamaterials with the 3D printing technology

**DOI:** 10.1038/s41598-019-52705-5

**Published:** 2019-11-07

**Authors:** Guan-Hua Li, Yi-Ze Wang, Yue-Sheng Wang

**Affiliations:** 1 Institute of Engineering Mechanics, Beijing Jiaotong University, Beijing, 100044 China; 2 Department of Mechanics, Tianjin University, Tianjin, 300350 China

**Keywords:** Mechanical engineering, Applied physics

## Abstract

Propagation of elastic waves along a direction has special interests in practical applications. These concerns generate the design of an elastic wave metamaterial with electrically switchable properties, which is studied in this work. The structure contains a T-shaped waveguide in a plate with the 3D printing technology; and the active control system is used to tune the propagation direction of the flexural wave. The piezoelectric patches which are connected by the negative capacitance circuits are applied to behave as the active control system. The finite element simulation is performed to give the theoretical prediction of the switchable waveguide and the tunable equivalent parameters are achieved by the electrical circuits. The active control experiments are finally carried out to support the numerical design.

## Introduction

During the past decades, phononic crystals and elastic wave metamaterials which have the periodic characteristics have received lots of attention^1–8^. This is mainly because that these periodic systems have superior properties, such as the wave band gaps^9–12^, negative refraction^13–16^, acoustic cloaks^17–19^, etc. These behaviors can be used to tune the wave propagation. Acoustic couplers^20^, sensors^21^ and waveguides^22^ are several typical applications of phononic crystals and elastic wave metamaterials.

Another important characteristic of phononic crystals and elastic wave metamaterials is the defect state. It’s well known that if there are some defects in these periodic structures, the elastic wave will be localized near the defect (for the point defect) or propagate along the defects (for the line defects). So the propagation direction and some other properties of elastic waves can be controlled by designing different waveguides. For instance, Rostami-Dogolsara *et al*.^23^ presented the switchable acoustic demultiplexers based on the fluid-fluid phononic crystals. Zhang *et al*.^24^ used the supercell plane wave method to investigate defect modes by introducing the bend-shaped linear defects. Wang *et al*.^25,26^ discussed wave guides with the reconfigurable and coupled-resonator properties. Yao *et al*.^27^ presented the flexural waves in the phononic crystal plate with linear defects to show the guide behaviors.

In recent years, special attention has been focused on the active control effects on phononic crystals and elastic wave metamaterials, which can show many interesting results^28–32^. In order to derive the broadband control of elastic waves in a flexible plate, Tateo *et al*.^33^ presented an experimental investigation about a periodic array of shunted piezoelectric patches with the negative capacitance. Casadei *et al*.^34^ discussed about the experimental demonstration of a tunable acoustic waveguide which was implemented on a phononic plate. By connecting gradient negative capacitance circuits to an array of piezoelectric patches, Chen *et al*.^35^ designed a metamaterial-based sensing system with the gradient bending stiffness. They demonstrated that the proposed system can achieve more than two orders of magnitude amplification of flexural wave signals to overcome the detection limit. In this work, we propose an elastic wave metamaterial with a T-shaped switchable waveguide by the active control system. With the electrical tunability, the switchable properties of the elastic wave are realized.

On the other hand, compared with the traditional materials, phononic crystals and elastic wave metamaterials have unique band gap characteristics. At present, they have shown broad prospects in waveguides, filters, new vibration and noise reduction materials and other devices. Phononic crystals and elastic wave metamaterials have become the hot research topic in many fields, such as engineering mechanics, physics, mechanical engineering, etc. The research in this field has attracted extensive attention and has broad development prospects.

Nowadays, the 3D printing technology has gradually replaced the traditional manufacturing technology. It’s mainly due to that the 3D printing technology can make most structural shapes with the precise size at different scales. As the structure becomes more complex by this method, the manufacturing cost will not increase. The 3D printing technology offers the opportunity for the traditional design and control of periodically patterned structures. And even it can realize some special characteristics, e.g. the negative stiffness and Poisson’s ratio^36^. So based on the 3D printing technology, special phononic crystals and elastic wave metamaterials would be achieved.

## Results and Discussions

In this section, the active control system is bonded on an elastic wave metamaterial plate which consists of the piezoelectric patches and negative capacitance circuits. Phononic crystals and elastic wave metamaterials have band gaps, in which elastic waves cannot propagate. When the elastic metamaterials have the defect like the point and line cases, the elastic wave will be limited to the point defect or propagate along the line defect. Phononic crystals and elastic wave metamaterials are the artificial structures with the periodic elastic constants and densities. Negative capacitance circuits can change the inherent elastic modules, which results in tuning the band gap structure. Then the elastic waves in specific frequencies can be controlled to propagate along the direction as we design. Then the propagation direction of elastic waves will be changed actively. Based on the finite element method, switchable properties of elastic waves in the metamaterial plate will be presented and the validity of numerical results will be verified by the experiment.

### Negative capacitance circuit

Assume that the polarization direction is along the *z*-axis, and the *x*, *y* and *z* directions are described as 1, 2 and 3, respectively. The piezoelectric equation can be expressed as^37^

1 $$\begin{array}{c}{S}_{1}={s}_{11}^{E}{T}_{1}+{d}_{31}{E}_{3}\\ {D}_{3}={d}_{31}{T}_{1}+{\varepsilon }_{33}^{T}{E}_{3}\end{array}$$

where *S*_1_ and *T*_1_ denote the strain and stress along the *x* direction; ${S}_{11}^{E}$ is the compliance coefficient of the piezoelectric material; *d*_31_ and ${\varepsilon }_{33}^{T}$ are the piezoelectric and dielectric constants; *E*_3_ is the electric field intensity along the *z* direction; and *D*_3_ is the electric displacement.

Substituting the expressions of the electric displacement and electric field into Eq. (1), the relation between the strain and stress becomes^38^

2 $${S}_{1}=({s}_{11}^{E}-\frac{sZ{d}_{31}^{2}{A}_{s}{h}_{p}^{-1}}{1+sZ{C}_{p}}){T}_{1}$$

where *s* is Laplacian operator; *Z* is the complex impedance of the shunting circuit; ${C}_{p}={\varepsilon }_{33}^{T}{A}_{s}{({h}_{p})}^{-1}$ is the inherent capacitance of the piezoelectric patch; *A*_*s*_ is the electrode area; and *h*_*p*_ is the patch thickness.

Then the inherent elastic modules of the patch can be derived by Eq. (2) as

3 $${E}_{p}=\frac{{h}_{p}(1+sZ{C}_{p})}{{h}_{p}{s}_{11}^{E}(1+sZ{C}_{p})-sZ{d}_{31}^{2}{A}_{s}}$$

**Figure 1:**
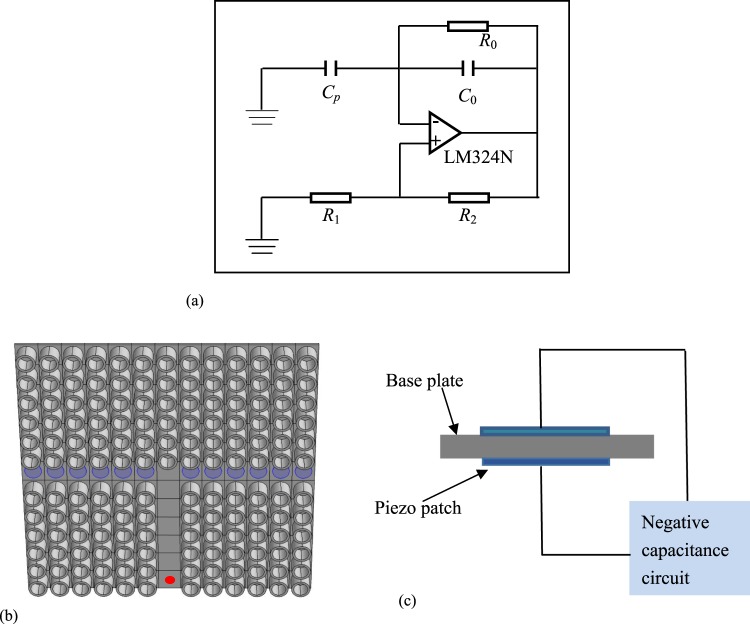
(**a**) Negative capacitance circuit. Finite element model: (**b**) the elastic metamaterial plate with a T-shape waveguide; and (**c**) the active unit cell with a piezoelectric patch attached by the negative capacitance circuit.

As shown in Fig. 1(a), the negative capacitance circuit is used in the active control system to change the elastic modulus, in which the capacitance *C*_*p*_, compensation resistance *R*_0_, operational amplifier, fixed resistances *R*_1_ and sliding rheostat *R*_2_ are included. In the experiment, the operational amplifier LM324N is applied and the complex impedance *Z* can be defined as

4 $$Z=\frac{1}{-\alpha {C}_{p}s}$$

where *α* = (*R*_2_ × *C*)/(R_1_ × *C*_*p*_).

Therefore, we can tune the value of *α* to change the inherent elastic modulus of piezoelectric patches and perform as the active control action on the elastic wave.

### Numerical simulation

The periodic structure consists of a thin resin plate with 5 mm thickness and the periodic arrangement of surface-bonded hollow cylinders. The external diameter of the hollow cylinder is 34 mm and internal diameter is 26 mm, as well as the height of the hollow cylinder is 30 mm. These cylinders are arranged as the square lattice with the constant 40 mm. As shown in Fig. 1(b), a T-shaped waveguide is designed by a 13 × 13 lattice and removing some cylinders. The horizontal channel is bonded by the circular P-4 piezoelectric patches with the diameter being 30 mm and the thickness being 1 mm. As shown in Fig. 1(c), each patch is connected with a negative capacitance circuit to behave as the active control load.

The structural optimization by the optimization method, such as the genetic algorithm, is not performed. During the preparation of this work, several different kinds of the periodic structures are compared. Such as cuboid columns, solid and hollow cylinders are arranged as the square lattice and we find that the periodical structure with hollow cylinders can show the satisfied result. Then different structural parameters are considered to improve the model. We know that it’s not the periodic structure with the best effect. It should be noted that in this work, the active control can show more importance.

The propagation properties of the elastic waves in the periodic structure are simulated by the finite element software COMSOL. As shown in Fig. 1(b), the flexural displacement is denoted by the red point to excite the elastic wave. Piezoelectric patches are attached to the left and right sides of the wave guide to generate the waveguide structure, which leads to band gap characteristics for both the right and left sides. We know that the elastic waves at some special frequencies will be localized near the defects of phononic crystals and metamaterials. This is the purpose to design this structure and the responses corresponding to the frequency from 0 to 6000 Hz are simulated by the finite element software. It can be found that the elastic wave is localized in the waveguide at 5390 Hz. A notable process is changing the propagation direction of the elastic wave by the external active control system. Then the negative capacitance circuits are connected by the right side of the horizontal channel and the parameter *α* = 0.9 is applied.

**Figure 2:**
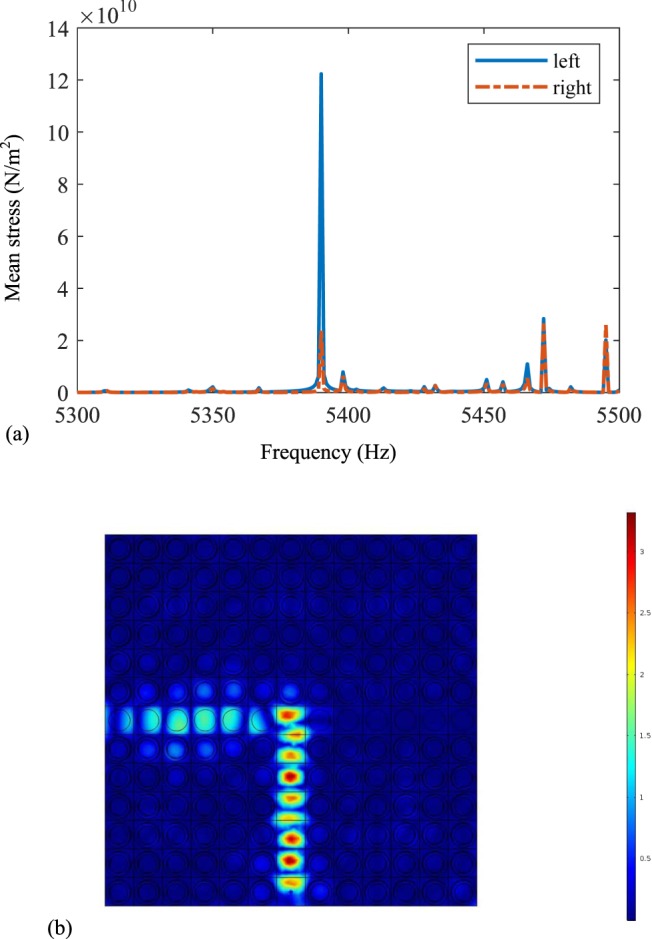
The switchable waveguide when the negative capacitance circuits are connected by the left side of the horizontal channel: (**a**) the mean stress of the left and right from 5300 Hz to 5500 Hz, and (**b**) the wave mode at 5390 Hz.

As shown in Fig. 2(a), the mean stress of the left and right sides are calculated as the elastic wave frequency changes from 5300 Hz to 5500 Hz. The solid and dotted lines represent the mean stresses on the left and right sides. We can see that when the wave frequency is 5390 Hz, the mean stress on the left side is much larger than that on the right side. According to the corresponding wave mode in Fig. 2(b), we can obviously find that the propagation of the elastic wave turns left when it arrives at the bifurcation point, and the elastic wave cannot propagate along the right side. It’s mainly because that the negative capacitance circuits change the equivalent elastic modules of the left-sided waveguide and the band structure will be changed. Then the line defect is formed, which can behave as the waveguide. Then the direction of the elastic wave will change and it propagates along the left side at the frequency 5390 Hz.

**Figure 3:**
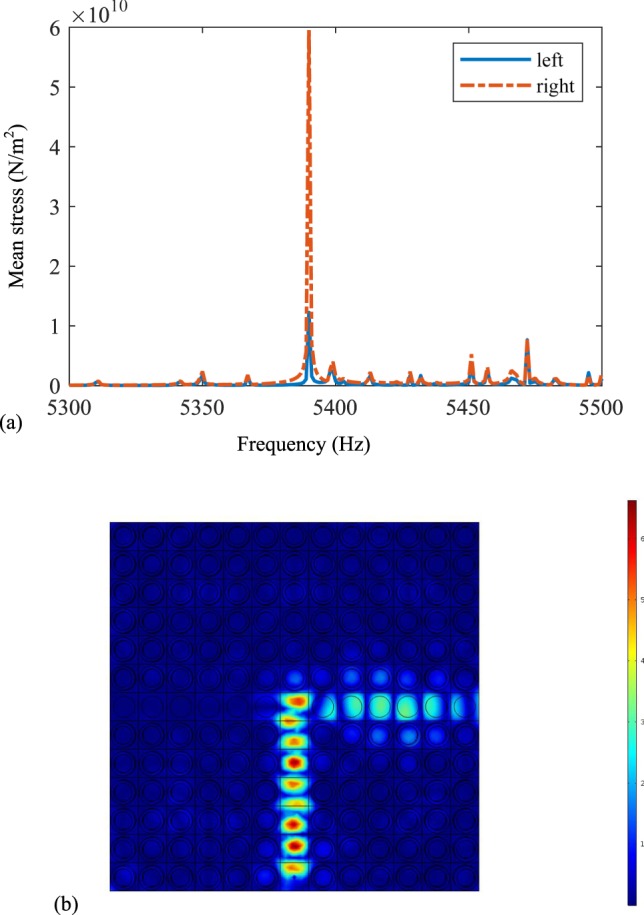
The switchable waveguide when the negative capacitance circuits are connected by the right side of the horizontal channel: (**a**) the mean stress of left and right from 5300 Hz to 5500 Hz, and (**b**) the wave mode at 5390 Hz.

In order to compare these results, the negative capacitance circuits are applied on the left side of the horizontal channel. The mean stress of the left and right sides with the same frequency range from 5300 Hz to 5500 Hz are simulated, and the results are shown in Fig. 3(a). The solid and dotted lines denote the mean stresses on the left and right sides. Then the similar conclusion can be obtained that the mean stress on the right side is much larger than that on the right one. From the wave mode distribution in Fig. 3(b), we can see that the elastic wave turns right when it arrives at the bifurcation, and cannot propagate along the left side when the frequency is 5390 Hz. Due to the line defect appears at this frequency, which can bound the propagation of the elastic wave. So the elastic wave will change the direction and propagate along the right side. Then the active control of the elastic wave metamaterials on the switchable properties is achieved.

### Experimental setup

To demonstrate the validity of the numerical simulations, we carry out the active control experiments on the waveguide structure, in which the P-4 piezoelectric patches are boned at the line defect. The waveguide structure is fabricated by the 3D printing technology. The Young’s modulus *E* = 2.5 GPa, the density *ρ* = 1300 kg/m^3^ and the Poisson’s ratio *ν* = 0.41. The material parameters of the P-4 piezoelectric patch are shown in Table 1.

**Table 1:** The material parameters of the piezoelectric patch.

Material	Mass density *ρ* (kg/m^3^)	Young’s modulus *E* (N/m^2^)	Compliance coefficient ${S}_{11}^{E}$ (m^3^N^−1^)	Piezoelectric strain coefficient *d*31 (Cm^−2^)	Permittivity ${\varepsilon }_{33}^{T}$ (Fm^−1^)
p-4	7450	8.83 × 10^10^	1.2 × 10^−11^	−1 × 10^−10^	1.2 × ^−8^

**Figure 4:**
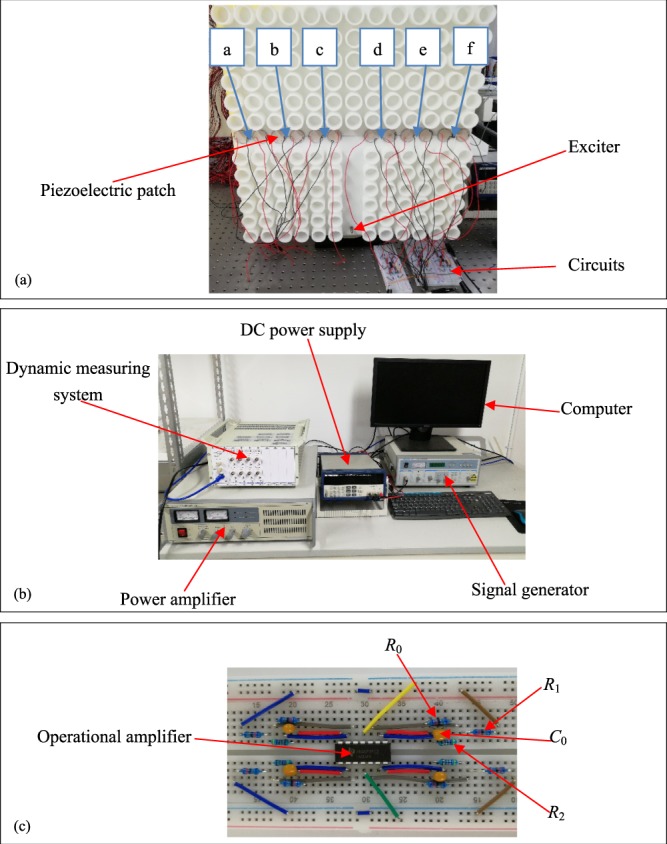
Experimental setup: (**a**) The switchable waveguide with the active control system, (**b**) the testing system and (**c**) the negative capacitance circuit.

The experimental setup is illustrated in Fig. 4. The waveguide structure is connected with the negative capacitance circuits. The elastic wave exciter and the collected points a-f are shown in Fig. 4(a). Figure 4(b) illustrates the experimental testing system, in which the signal is generated and the responses are received and saved in the computer. As shown in Fig. 4(c), the DC power supply depends on the operational amplifier, which is an important device in the negative capacitance circuit. The explicit description of the circuit is listed in Table 2.

**Table 2:** The parameters of the negative capacitance circuit.

*C* (*pF*)	*C*_*p*_ (*pF*)	*R*_2_ (kΩ)	*R*_1_ (kΩ)	R_0_ (kΩ)	Operational amplifier
9.684	8.155	51.54	68	2000	LM324N

**Figure 5:**
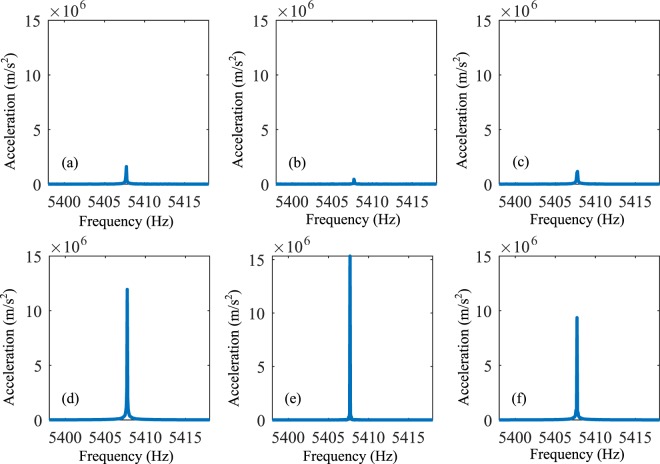
Responses of points a-f at 5408 Hz when the left side of the horizontal channel is exerted by the active control system.

**Figure 6:**
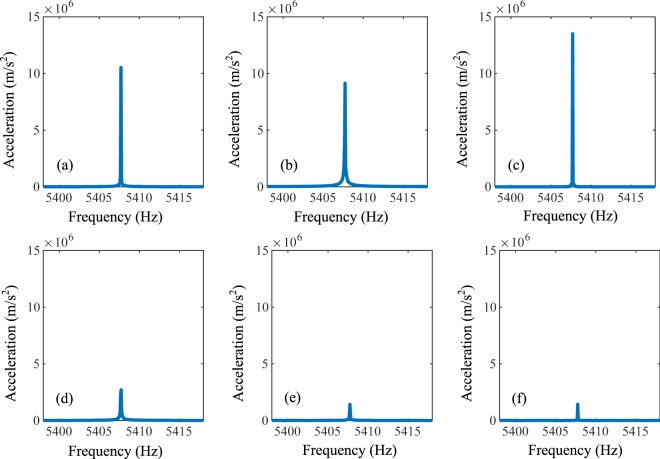
Responses of points a–f at frequency at 5408 Hz when the right side of the horizontal channel is exerted by the active control system.

Figure 5 and 6 show the experimental results, in which the subsets (a–f) correspond to the responses of the points a–f. Considering the connecting circuits on the left side of the horizontal channel, we can see that the responses of the points a–c are much smaller than those of the points d–f at 5408 Hz. It means that at this frequency, most energy of the elastic wave propagates along the right side. Moreover, if the negative capacitance circuits are connected by the right side of the horizontal channel, we can find that the responses of the points a–c are much larger than those of the points d–f at 5408 Hz. It can be concluded that the elastic wave can hardly transmit along the right side but propagate along the left side very well.

We know that the experimental testing system cannot present the displacement distribution field like the numerical results. It can test the acceleration signal at a certain point. So we set three receiving points for the acceleration signal on the left or right side to get the information about the wave propagation. When the acceleration signals of the three points on the left are much higher than those on the right, we can assume that the elastic wave changes the direction and propagates along the left of the waveguide. On the other hand, similar behaviors can also be derived if the wave propagates along the right side. These acceleration responses are shown in Figs 5 and 6. Then, the response for the frequency at 5408 Hz appears a satisfied result which is close to the numerical simulation for the frequency being 5390 Hz. As a result, the active control of the wave propagation can be supported well by the experiments.

## Conclusions

Previous investigations on the elastic waveguides are mainly concentrated on the propagation of elastic waves only along a single direction. In this work, the switchable waveguide of the elastic wave metamaterials is achieved by the active control action. In order to control the material parameters, the switchable waveguide is connected with the piezoelectric patches which are attached by the negative capacitance circuits. Furthermore, the experimental model is fabricated by the 3D printing technology and bonded by the active control system. The validity of the numerical simulation is supported by the experiment. This study is expected to be helpful to design the elastic wave metamaterials with the active control action.
